# Shaping the future of Leptospira serotyping

**DOI:** 10.1099/jmm.0.002059

**Published:** 2025-09-12

**Authors:** Alexandre Giraud-Gatineau, Kouessi Dagbo, Helena Pětrošová, Catherine Werts, Fréderic J. Veyrier, Mathieu Picardeau

**Affiliations:** 1 Biology of Spirochetes Unit, Institut Pasteur, Université Paris Cité, Paris, France; 2 Bacterial Symbionts Evolution, Centre Armand-Frappier Santé Biotechnologie, Institut National de la Recherche Scientifique, Université du Québec, Laval, Canada; 3 Genome BC Proteomics Centre, University of Victoria, Victoria, BC, Canada; 4 University of Victoria, Victoria, BC, Canada; 5 Biology and Genetics of Bacterial Cell Walls unit, Institut Pasteur, Université Paris Cité, Paris, France

**Keywords:** diagnosis, epidemiology, leptospirosis, serology, strain typing

## Abstract

Leptospirosis is a re-emerging zoonosis caused by a diverse range of pathogenic *Leptospira*, which are divided into species, serogroups and serovars. Although advances in genomics have recently refined species classification, serotyping, which is based on the antigenic variability of lipopolysaccharides O-antigens, still relies heavily on traditional and labour-intensive methods. In addition, the molecular basis of serovar diversity is not fully understood, which poses challenges for rapid and accurate serovar and/or serogroup identification. However, identification of serovars remains crucial for epidemiological studies, surveillance, diagnostics, understanding host–pathogen interactions and vaccine development. In this review, we assess current techniques for *Leptospira* serovar and serogroup identification and explore emerging DNA-based methodologies for serovar and serogroup prediction.

## Introduction

Leptospirosis is a neglected zoonotic disease of global importance, commonly associated with rat-borne transmission, poverty, urbanization and climate change [1]. Leptospirosis causes over one million cases and nearly 60,000 deaths annually [2]. These numbers are likely underestimated because of undiagnosis, misdiagnosis and under-reporting, particularly in regions where other diseases with similar non-specific presentations, such as dengue and malaria, are prevalent [3].

Leptospirosis is caused by several species of pathogenic *Leptospira* and is transmitted to humans through exposure to soil or water contaminated with the urine of an infected animal, such as rats or other mammals. *Leptospira* can also be found in the environment as free-living saprophytes [4]. Within the *Leptospira* genus, which belongs to the phylum of spirochetes, bacteria share similar morphology, with only subtle biochemical differences distinguishing saprophytic from pathogenic species [5]. Historically, the limited availability of distinct phenotypic characteristics for classifying *Leptospira* isolates with significant epidemiological relevance made serology a cornerstone of leptospiral classification [4]. In the 1990s, the DNA–DNA hybridization methods emerged and the first *Leptospira* species were described [6,8]. The recent advent of next-generation sequencing and the subsequent boom of DNA sequencing technologies have redefined bacterial diversity with unprecedented resolution. Whole-genome sequencing (WGS) has become the new gold standard for bacterial species delineation [9]. Criteria for the identification of new *Leptospira* species have recently been revised and are available to the research community (https://leptosociety.org/resources/). The criteria include mandatory metadata, a defined threshold for DNA–DNA relatedness to the genomes of existing species, publication of the species description in a peer-reviewed journal and deposition of the strain in reference laboratory collections.

The genus *Leptospira* currently consists of 74 validated *Leptospira* species that are subdivided into 2 subclades of free-living non-pathogenic species (S1 and S2) and 2 subclades of species with variable pathogenic potential (P1 and P2) [10]. The P1 subclade can be further subdivided into two groups: the P1+group comprises eight species (*Leptospira interrogans*, *Leptospira kirschneri*, *Leptospira noguchii*, *Leptospira santarosai*, *Leptospira mayottensis*, *Leptospira borgpetersenii*, *Leptospira alexanderi* and *Leptospira weilii*), which possess the ability to evade the host immune system and cause severe disease in both humans and animals [11]. The P1 group includes species that, similar to those belonging to the P2 subclade, are mostly environmental isolates with low virulence [11]. Some P1+species are presumed to have different geographical distributions. For example, *L. interrogans* can be found all over the world, while others like *L. noguchi*, *L. santarosai* and *L. mayottensis* are restricted to specific regions [12].

Currently, molecular typing is widely used to identify *Leptospira* strains at the species and subspecies levels. Genotyping approaches vary, ranging from single-gene analyses [13,14] to multilocus sequence typing (MLST) using six to seven housekeeping genes [15,17], or broader genome-wide comparisons [18]. However, these methods often lack the resolution needed to discriminate between epidemiologically related strains, and no consensus exists on a standardized genotyping approach for global data comparability. Moreover, commonly used genotyping markers are not linked to the genes encoding the lipopolysaccharides (LPS), which play a key role in host adaptation and virulence as described above. The *rfb* cluster of genes, which encodes most LPS biosynthesis enzymes, can be horizontally transferred between species, further complicating any direct correlation between genotype and serovar or serogroup classification. As a result, current genotyping strategies fall short of providing a comprehensive epidemiological perspective. Identification of the serogroup and serovar thus remains a critical tool for epidemiological strain characterization. Following the recent revision of the nomenclature for the genus *Leptospira* to the species level [10], which was followed by the formal identification of numerous new species [5,19,21], it is now imperative to reevaluate the serological classification of *Leptospira* spp.

The LPS-based serological classification of *Leptospira* isolates remains widely used in the field, both due to historical precedent and its relevance to epidemiology and protective immunity [4]. Regardless of species, *Leptospira* strains have been classified into serovars based on the serological reactivity of their LPS, using the reference method known as the cross-agglutinin absorption test (CAAT). Serovars are further grouped into serogroups based on shared antigenic characteristics [4]. The *Leptospira* genus is thus divided into over 250 serovars belonging to 26 serogroups [22,23]. However, information on animal hosts is lacking for the majority of serovars [22]. It should be further noted that the majority of the new environmental species described in recent years have no assigned serovars [5,19,21].

**Fig. 1.**
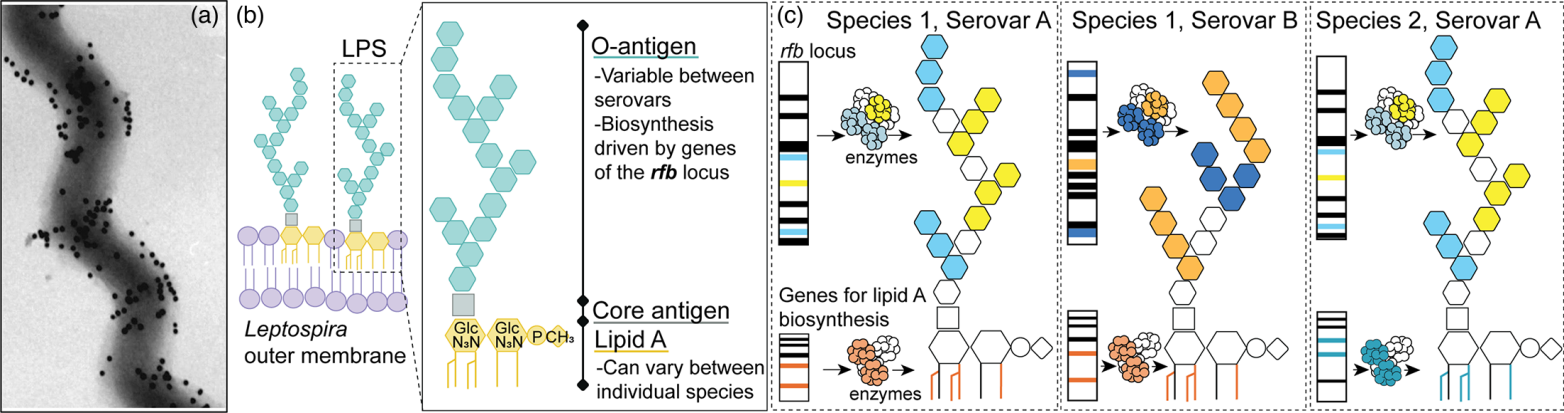
The *Leptospira* LPS. (**a)** *L. interrogans* coated with gold-labelled anti-LPS monoclonal antibodies [79]. (**b)** Schematic representation of *Leptospira* LPS. GlcN_3_N, di-aminoglucose; P, phosphate; CH3, methyl group.** (c)** Proposed model of *Leptospira* LPS diversity between species and serovars. Different species can belong to the same serovar. Vice versa, the same serovar can be found across multiple species.

As mentioned above, there is not always a consistent correlation between species and serovars. The same species can include multiple serovars, and the same serovar can be found across different species (Fig. 1) [4]. The resulting classification system, often not properly used in the scientific literature, requires a more precise and standardized approach to align with the updated taxonomic framework and enhance its utility in research, epidemiology and clinical diagnostics. This challenge was already highlighted over 40 years ago when Terpstra noted, ‘In the last few years, it has become increasingly clear that the serovar concept is no longer fully satisfactory as it may fail to adequately define epidemiologically important entities. In addition, CAAT is too cumbersome and time-consuming for routine typing’ [24]. The discontinuation of the CAAT reference technique, combined with the increased accessibility of WGS, should encourage the development and adoption of novel DNA-based methods for serovar prediction. Furthermore, it is essential to critically assess the epidemiological value of serovar identification and to question whether the term ‘serovar’ remains relevant in contemporary scientific discourse. This article aims to review the current state of the field and provide a foundation for informed discussion on these issues.

## *Leptospira* LPS and serovars

LPS is a major component of the outer membrane of Gram-negative bacteria and acts as a potent activator of the innate immune system, as well as a target of adaptive immune responses. However, among spirochetes, which are also diderms, only *Brachyspira* and *Leptospira* spp. possess LPS. Despite its importance in the pathogenesis of leptospirosis and innate immune evasion [25,27], the structural and functional properties of *Leptospira* LPS remain poorly characterized.

### LPS structure and diversity

LPS is composed of three components: the lipid A, which is anchored to the bacterial outer membrane, the core oligosaccharide and the O-antigen (Fig. 1). *Leptospira* spp. possess all genes required for lipid A biosynthesis. The structure of lipid A has been characterized in approximately one-third of the reference *Leptospira* species, corresponding to only a few known serovars [28,30]. Across all *Leptospira* characterized to date, the lipid A has only one methylated phosphate; all primary fatty acyl chains are linked to the disaccharide backbone by amide bonds and several unsaturated fatty acyl chains [31]. These unique structural characteristics likely contribute to the lipid A’s high stability and the ability of *Leptospira* to evade the host’s innate immune response. However, structural diversity also exists across *Leptospira* species, driven by the length, degree of unsaturation and the number of fatty acyl chains in the lipid A molecules. To date, ten different lipid A profiles were identified across the genus, with the strongest association observed between these profiles and classification of individual *Leptospira* species into P1+, P1-, P2, S1 and S2 groups [30]. Moreover, the lipid A portion of LPS appears mostly conserved within the individual species, regardless of their assigned serovar.

The O-antigen is the most surface-exposed component of the LPS (Fig. 1). The general chemical composition of the O-antigen has been characterized in some leptospiral isolates, but the precise structure remains unknown [25,32]. The O-antigen is composed of different monosaccharides, synthesized and oligomerized together by the action of several enzymes (Fig. 1). In *Leptospira*, most, if not all, of these enzymes are encoded within a genomic locus called the ‘*rfb* cluster of genes’ [33]. The size of the *rfb* cluster is variable and can reach more than 100 kb, with the P1+species harbouring larger and more complex compositions than their saprophytic counterparts [34]. Several studies suggest that the *rfb* cluster of genes encodes for the serovar [12,35,40].

### Role of LPS

*Leptospira* LPS is a critical component of the outer membrane of *Leptospira* and plays several essential roles as described below.

#### Host recognition of LPS

LPS is expected to play a key role in host-pathogen interactions as the outermost and most variable structure of the LPS; the O-antigen serves as the primary contact between *Leptospira* and the host immune system. In addition, *L. interrogans* mutants with defective LPS are attenuated in virulence [26,29]. In comparison to the canonical LPS from *Escherichia coli*, LPS from *L. interrogans* is less endotoxic [31]. Lipid A of *L. interrogans* does not act as a human TLR4 agonist [41], likely because of its peculiar structure [28]. However, *L. interrogans* LPS is recognized by mouse TLR4 [31], indicating species-specific differences in innate immune detection. Interestingly, *L. interrogans* LPS activates human and mouse TLR2, a response attributed to tightly bound lipoproteins that co-purify with the LPS during extraction [42]. Mice that are typically resistant to leptospirosis become susceptible when they have a defective TLR4, highlighting the importance of LPS recognition in disease pathophysiology. Susceptibility is further increased in mice lacking both TLR4 and TLR2 [43]. As stated above, *Leptospira* lipid A shows structural differences between P1+ (highly virulent pathogens), P1-, P2 and S (low-virulent pathogens or saprophytes) species [30]. These findings suggest that *Leptospira* lipid A may play a role in the distinct recognition of these species by host immune cells.

#### Host adaptation and disease severity

A wide range of mammalian species can be infected [22], resulting in asymptomatic carriage in natural reservoirs or maintenance hosts and acute infections in accidental hosts. Some serovars are thus adapted to specific animal host species. For example, rats infected with the *L. interrogans* serovar Icterohaemorrhagiae exhibit chronic, asymptomatic colonization of the kidneys, whereas this serovar causes severe infections in accidental hosts such as humans and dogs [44,45]. However, the distinction between maintenance and accidental hosts is not always clearly defined, as the interaction between pathogenic leptospires and animal hosts may be influenced by a variety of factors, including the host immune response, the infecting serovar, the inoculum dose and individual susceptibility. Despite the fact that large-scale studies will be needed to unravel these complex interactions, determining the infecting serovar is clinically significant, as some serovars, such as serovar Icterohaemorrhagiae, are known to be responsible for more severe infections in humans [46,49].

#### Protective immunity and vaccines

Naturally acquired protective immunity against leptospirosis is predominantly antibody-based and targets the immuno-dominant LPS structure, which varies across serovars [50,51]. The antibody response against LPS is mainly T-cell independent and does not induce immune memory. Current vaccines, consisting of inactivated whole-cell bacterins from the main circulating serovars, only provide short-term protection against a narrow spectrum of serovars, typically within a single serogroup [52]. Therefore, comprehensive serotyping and surveillance are essential to assess the efficacy of the vaccines used and to inform decisions on vaccine composition. Additionally, more work is needed on the characterization of LPS molecules as they exist in the host environment. The O-antigen from host-derived bacteria differs from that found in bacteria grown in the traditional EMJH medium [53]. The resulting difference in structure and its impact on immune recognition is unknown.

#### Diagnosis, epidemiology and surveillance

The gold standard for sero-diagnosis of leptospirosis, microscopic agglutination testing (MAT), relies on a panel of live *Leptospira* strains that should reflect the diversity of strains circulating in a given region. The test detects serogroup-specific antibodies present in the host serum (see below). In some cases, the characterization of the infecting strain at the serogroup level can provide information on potential animal hosts acting as sources of infection. Such information on the origin of the infecting strain can subsequently be used for control and intervention measures, including the development of new animal vaccines and reservoir control programmes.

## How to determine the *Leptospira* serotype

**Fig. 2.**
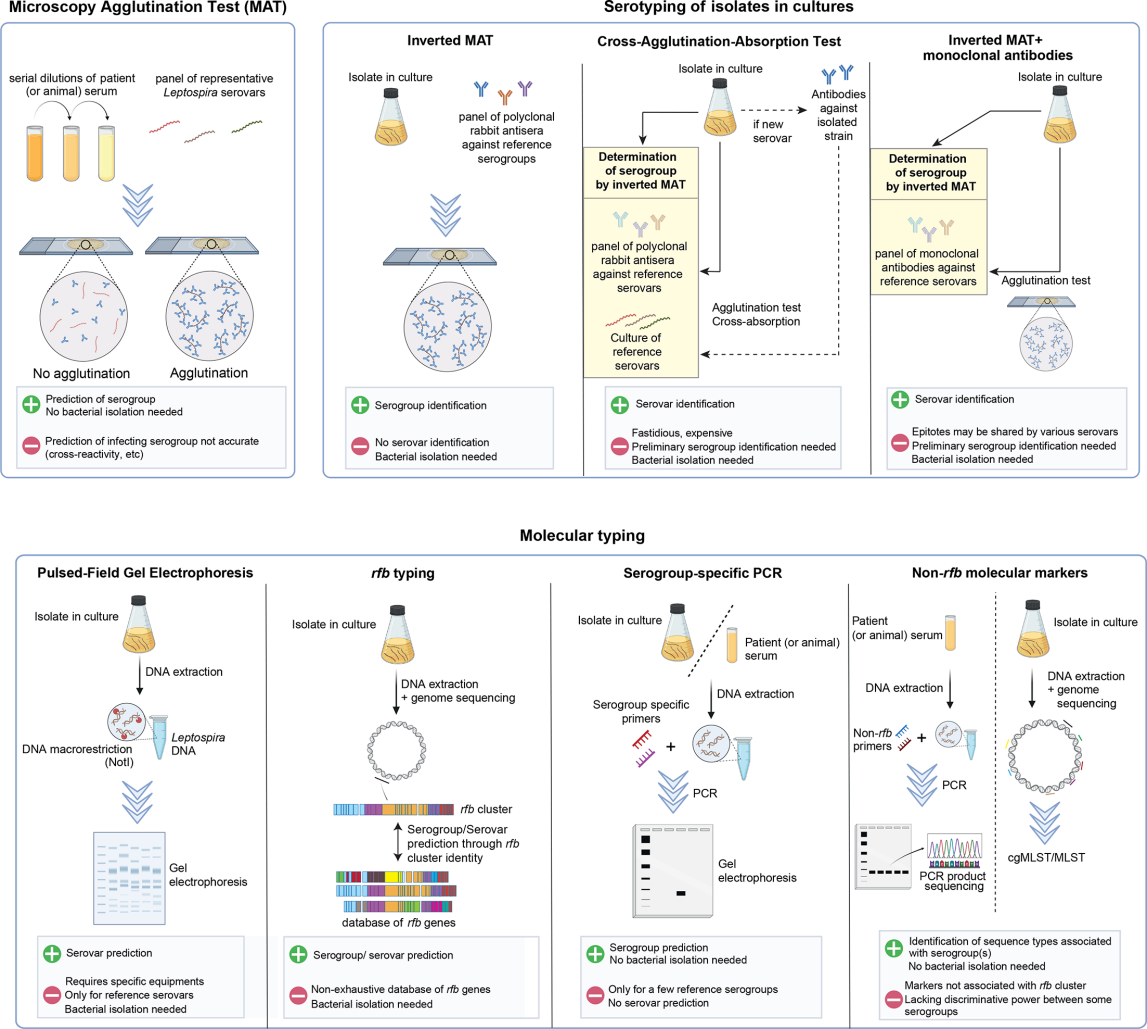
*Leptospira* serotyping methods. MAT performed on serum samples are not a reliable predictor of the infecting serogroup. Serotyping based on isolates requires expertise in traditional techniques and can be labour-intensive. Molecular typing, while promising, may require specialized equipment and access to genome sequencing. Moreover, these molecular approaches are still undergoing validation on a larger number of serovars.

Currently, several options are available for serotyping *Leptospira*, including both traditional and molecular methods. Each of the techniques presents distinct advantages and limitations that are discussed below (Fig. 2).

### Kinetics of infection and sample types

After an incubation period of 1 to 2 weeks, leptospirosis typically presents as a biphasic illness. The acute/bacteraemic phase occurs during the first week after the symptom onset, and the bacterium or its DNA can be detected in blood and urine. This phase is followed by antibody production in the late/immune phase, ~1 week after the symptom onset, during which the organism is no longer circulating in the blood but may persist intermittently in the urine [4]. Therefore, culture and DNA-based methods are most effective in the acute phase of the disease. In the convalescent phase, serological testing combined with culture and DNA-based methods using patients’ urine is the only means of attempting to identify the infecting serovar and serogroup.

### Prediction of infecting serogroup from MAT

The MAT developed by Martin and Pettit over a century ago [54] remains the serological reference test for the diagnosis of leptospirosis. Detectable agglutinating antibodies appear in the blood 1 week after the onset of symptoms and can persist for several months. The test consists of exposing serial dilutions of patient (or animal) serum to live *Leptospira* strains representing locally circulating serovars, followed by dark-field microscopy to measure the extent of agglutination caused by serum antibodies binding to each representative serovar. As an example, the French National Reference Center for Leptospirosis is using a panel of 24 isolates belonging to 22 and 24 different serovars and serogroups, respectively (Table S1, available in the online Supplementary Material). The highest serum dilution that agglutinates 50% or more of the leptospires is considered the antibody titre. In principle, MAT can identify the infecting strain at the serovar level, but its resolution is insufficient to discriminate between serovars belonging to the same serogroup. In addition, several studies have shown that the highest antibody titre is not a reliable indicator of the infecting serogroup and overinterpretation of titre values can lead to misidentification [55,59] (Table 1). There are many reasons for inconclusive serogroup prediction using the MAT:

1. Cross-reactivity. Anti-*Leptospira* antibodies generated by infection with one serovar can react with antigens of multiple serovars across serogroups. This is especially true in the early stages of infection.
2. Paradoxical reactions. In acute and early-convalescent phases of infection, the highest MAT litres can be detected against a serovar unrelated to the infecting one, due to heterologous reactions being stronger than homologous reactions.
3. Inter-laboratory variability in MAT data. Variability in titre magnitudes has been documented across reference laboratories [60]. This may be due to the panel of strains used, testing conditions and subjectivity of evaluation of the degree of agglutination by the personnel.
4. Antibiotic therapy. The antibody response may be delayed or reduced if antibiotic therapy was given before MAT.
5. MAT reactivity in different host species. MAT cross-reactivity profiles and titre levels can vary in different animal host species, as shown in a recent study in foxes, sea lions and skunks infected with the same serovar [59].

**Table 1.** Summary of studies demonstrating discrepancies between MAT results and the serogroup of the infecting strain These findings show that the serogroup corresponding to the highest MAT antibody titre does not reliably reflect the actual infecting serogroup.

No. of isolate	Distribution of serogroup	Country and year period	Sensitivity of MAT on patient sera for the prediction of infecting serogroup (%)*	Reference
151	Autumnalis (65%), Icterohaemorrhagiae (23%), Ballum (11%), Australis (1%)	Barbados, 1980–1998	44	[57]
106	Autumnalis (86%), Pyrogenes (7%), Javanica (3%), Bataviae (1%), Grippotyphosa (1%), Pomona (1%), Sejroe (2%)	Thailand, 2000–2006	33	[55]
52	Icterohaemorrhagiae (89%), Canicola (4%), Autummnalis (2%), Sejroe (2%) and Grippotyphosa (2%)	Brazil, 1995–2010	94	[58]

*Based on the highest MAT titre.

In conclusion, MAT titre results can be influenced by multiple factors. In the absence of supporting evidence, MAT results alone must be interpreted cautiously for the identification of the presumptive serogroup. Definitive conclusions about infecting serovar cannot be drawn based on MAT without bacterial isolation.

### Serotyping of cultured isolates

Culture isolation is challenging but remains the gold standard for complete strain identification at the levels of species, serogroup and serovar. New culture media have been developed to prevent contamination and support the primary isolation of fastidious pathogenic leptospires, making it easier to isolate and cultivate strains [61,62]. Once isolation has been achieved, the strain can be fully characterized at the serovar and/or serogroup level using various approaches listed below:

#### i) Serogrouping using rabbit antisera (inverted MAT)

The serogroups are usually determined using a panel of polyclonal rabbit antisera representing all individual serogroups. Similar to MAT, the degree of agglutination of a strain suspension of unknown serogroup is estimated for each immunoserum. If the reaction is positive (less than 50% free bacteria), the titre is then determined by serial dilutions of the rabbit antiserum. The serogroup is assigned according to the antiserum that gave the highest agglutination titre [63]. An unknown serovar or serogroup should not agglutinate, or agglutinate to a lower extent, with any of the antisera.

#### ii) Serovar identification using CAAT

CAAT is the gold standard method to determine the serovar [23,64]. This procedure is complicated, involving several steps, time-consuming and is currently performed exclusively by the reference centre in Amsterdam (https://leptospira.amsterdamumc.org/leptospirosis-reference-centre/).

First, the serogroup is identified by subjecting a strain with an unknown serovar to inverted MAT (see above). Secondly, cross-agglutination is performed to identify the serovar by comparing the reactivity of the unknown strain with that of reference serovars within the identified serogroup. The ratio of heterologous to homologous MAT litres is then calculated: the homologous titre is obtained using a reference serovar and its antiserum, while the heterologous titre is measured by testing the reference antiserum against the unknown strain. A ratio above 10% indicates sufficient antigenic similarity to warrant the inclusion of that reference serovar in absorption testing [65]. In the third step, absorption tests are performed by incubating the reference antisera with the unknown strain. After absorption, the supernatant (absorbed antiserum) is tested again by MAT to determine whether antibodies specific to the homologous strain remain. A residual titre of ≥10% of the original indicates that the two strains are antigenically distinct (i.e. different serovars), while a reduction to <10 % suggests identity. If none of the tested reference serovars match the unknown strain (due to an absence of sufficient reactivity in the initial MAT or a lack of suitable absorption targets), the strain is considered a novel serovar. Rabbit antiserum must then be generated against the unknown strain, and the CAAT procedure is repeated to formally assign the new serovar.

#### iii) Serovar identification using monoclonal antibodies

Alternative methods using monoclonal antibodies have been described. After the identification of the serogroup using inverted MAT, isolates can be further typed at the serovar level by performing MAT with panels of monoclonal antibodies (mAbs) that characteristically agglutinate serovars from the serogroup of interest. The reference centre in Amsterdam is providing monoclonal antibodies produced after immunization of mice with the main reference serovars (https://leptospira.amsterdamumc.org/leptospirosis-reference-centre/typing-with-monoclonal-antibodies/). Most serovars, but not all, can be identified by their characteristic antigenic patterns recognized by a set of monoclonal antibodies, typically represented as histograms. However, results obtained using mAbs may be inconsistent with those produced by CAAT, as the epitopes recognized by mAbs can be shared among multiple serovars [66].

#### iv) Deducing the serotype from genes, loci and genomes

##### Serovar identification using PFGE

In the 2000s, some laboratories used PFGE for serovar prediction as genomic DNA macrorestriction analysis using NotI enzyme correlated well with serovar identity [67,69]. However, this method requires specific equipment not affordable to all. In addition, only the most common serovars have been typed, and the interpretation and comparison of PFGE profiles is not straightforward.

##### Serovar prediction using the *rfb* cluster

**Fig. 3.**
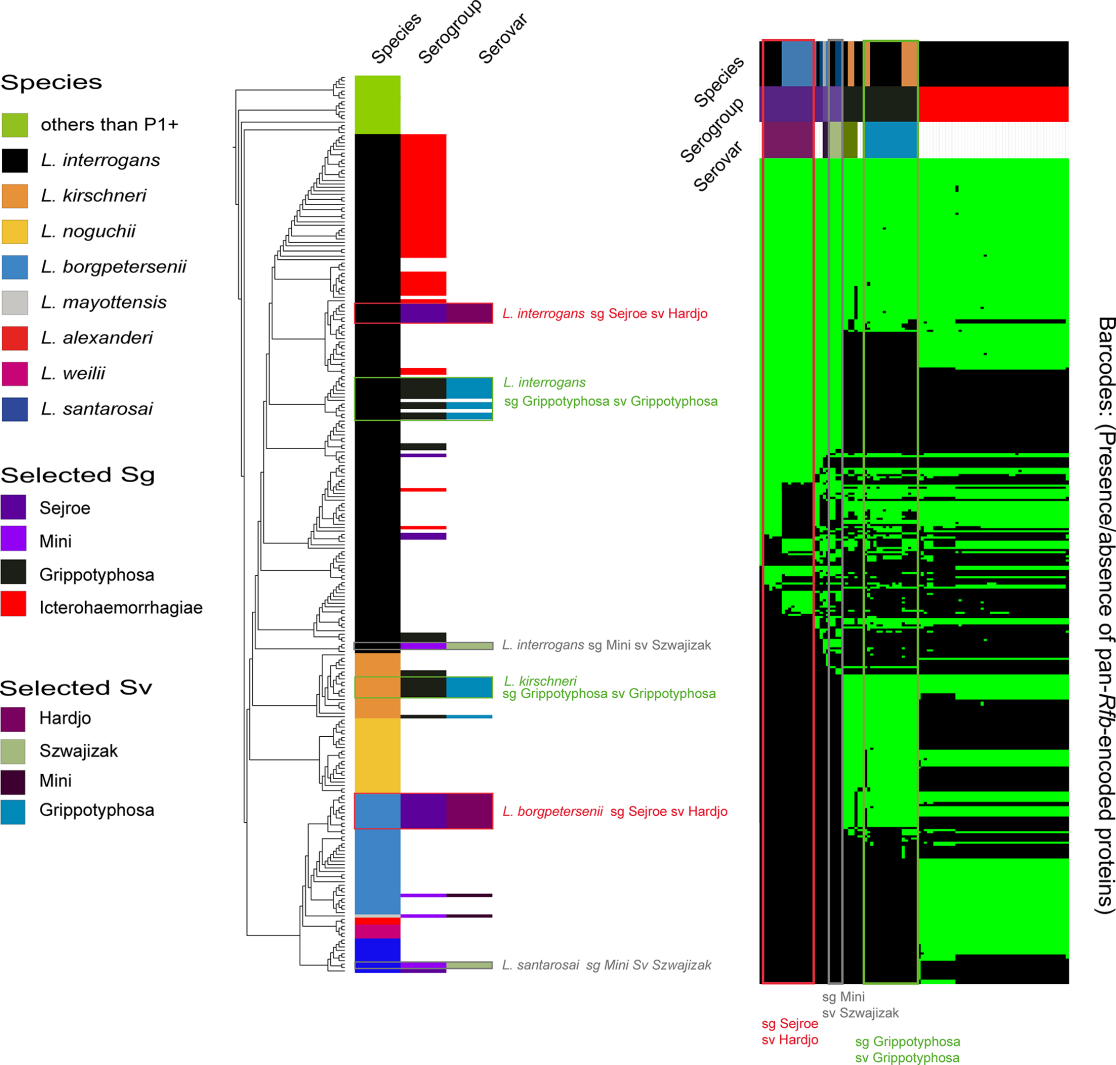
Phylogenetic distribution and barcodes of selected multi-species serogroups and serovars. This figure presents a subset of the *Leptospira* genomic database for which serovar and serogroup classifications have been determined. (**a**) Core-genome phylogeny of this genomic subset, illustrating the distribution of genomes by species. The associated serogroups (sg) and serovars (sv) are indicated, showing that some serogroups and serovars occur in multiple species. For simplicity, except serovars of serogroup Icterohaemorrhagiae that are represented to emphasize their important occurrence, serovars found exclusively in a single species are not shown. (**b**) Barcode profiles, representing the presence or absence of specific proteins encoded in all reference *rfb* clusters (pan-rfbs), as previously described [40], are organized using a dendrogram based on these presence–absence patterns. Again, for simplicity, only barcodes for multi-species serogroups and serovars and Icterohaemorrhagiae are presented. Here, barcodes cluster according to serogroup and serovar rather than by species, emphasizing the distinction between species classification and serogroup/serovar prediction.

The *rfb* cluster, which encodes genes involved in the biosynthesis of the O-antigen, displays variability in chromosomal position, in gene composition and in length. In recent years, several studies have shown that the gene composition of the *rfb* cluster strongly correlates with *Leptospira* serovar designation [12,38,40] and can, therefore, be used to design tools to predict serovars. In the future, a serovar-specific genetic fingerprint based on *rfb* genes could be instrumental in shifting from traditional serological techniques to molecular typing (Fig. 3). Literature [34] and bioinformatics analyses consistently show that the *rfb* cluster of P1+*Leptospira*, averaging 100 kb in length, is characterized by the presence of three hallmark genes: *marR* which encodes a transcriptional regulator (one copy) at the beginning of the locus, the *GDP-l-fucose-synthase* gene (two to four copies) within the locus and *sdcS* which encodes a *sodium/sulphate symporter* (one copy) at the end of the locus. By obtaining the positions of these genes within the same contig of a genome from second- or third-generation sequencing data, it is possible to extract the *rfb* cluster and create a reference database of unique group of *rfb* cluster encoded proteins sequences from multiple representative *Leptospira* strains [12,38,40] (Fig. 3). This representative set of strains comprises genomes with non-fragmented *rfb* clusters and will require further refinement to add diversity and include multiple isolates from the maximum amount of formally assigned serogroups and serovars. A possible limitation is that some highly related serovars could be difficult to discriminate using this method. This is the case of the serovars Icterohaemorrhagiae and Copenhageni, as no difference in the *rfb* could be strictly linked with the serovar. Santos et *al*. have identified a frameshift mutation within a homopolymeric tract of the *lic12008* gene in all the *L. interrogans* serovar Icterohaemorrhagiae strains but not in the Copenhageni strains [70]. To enhance the level of discrimination, one could incorporate this protein sequence into the set of a unique group of homologous proteins present in the reference database of *rfb*s. Of note, as of today, most *Leptospira* genomes are highly fragmented, preventing the assembly of the complete *rfb* cluster. In addition, the determination of *rfb* cluster boundaries in the P2, S1 and S2 sub-clades is not defined. Therefore, although this method is promising, it will necessitate validation across a wide range of serovar genomes to accurately define the cutoff for assigning genomes to specific serovars. Ideally, we will soon have an easily accessible programme that allows accurate serovar prediction by submitting a genome and comparing it with a curated database.

##### Serogroup-specific PCR

Primers targeting the *rfb* cluster could provide a rapid and specific molecular approach for identifying *Leptospira* serogroups and serovars. This approach has the advantage of allowing DNA amplification from biological samples without the need for culture isolation. Recent studies have demonstrated that specific primers can effectively distinguish between major pathogenic *Leptospira* serogroups [71,73]. However, there are notable limitations to *rfb*-based PCR. First, not all serogroups nor all serovars within a given serogroup have been tested by *rfb*-based PCR. Second, while the primers effectively distinguish some serogroups, they often fail to differentiate between serovars due to recombination events and structural rearrangements within the *rfb* region. This limitation is particularly important in cases where novel or less-characterized serovars or even serogroups are involved, leading to false-negative results. Finally, the effectiveness of *rfb*-targeting PCR is highly dependent on the availability of well-annotated genome sequences for reference. Without continuous updates to genomic databases associated with bacterial isolation, the accuracy of serogroup classification may be compromised, particularly for newly emerging or rare *Leptospira* strains.

##### Serovar identification using non-*rfb* molecular markers

Sanger sequencing of PCR products has been used for typing of isolates at the subspecies level using various genes, including *secY* and *lfb1* [13,14]. Again, PCR-based methods are rapid, technically simple and suitable for direct genotyping of strains present in biological samples without the need for culture isolation. However, the target genes are not associated with the locus involved in the determination of the serovar (i.e. the *rfb* cluster, see above). The discovery of short DNA sequence repeats in the genomes of *Leptospira* has led to the development of multiple loci variable number of tandem repeat analysis, but this method is restricted to a few species and has not been validated for all serovars [74]. Similarly, genus-specific core genome MLST and pathogen-specific MLST schemes, based on non-serovar-determining genes, can subdivide species into clonal groups (CGs). However, there is no strict correlation between CGs and serogroups and serovars, as isolates from the same serogroup or serovars can be distributed in different CGs [15,16, 18, 75]. Furthermore, these methodologies have not yet been applied across the full diversity of *Leptospira* isolates. Therefore, they may not detect the given serovar in all the species that harbour this serovar. To conclude, evidence suggests that PCR-based molecular typing for serovar prediction should be based on analysis of the *rfb* cluster.

### Current and possible future nomenclatures

The genus *Leptospira* is divided into 74 validated species, each of which is further divided into numerous serovars and serogroups based on serological testing. The accepted nomenclature is genus name, followed by species name, followed by serovar or serogroup, followed by strain (if appropriate), for example, *Leptospira* (genus name, italics, capital letter) *interrogans* (species name, italics, lower case) serovar Icterohaemorrhagiae (non-italics, capital letter) strain Verdun (non-italics, capital letter). However, with the advent of molecular typing, the nomenclature will likely evolve to contain sequence types, clonal groups, species groups and genovars, which could refer to groups of strains with similar *rfb* clusters of genes until their correspondence to a known specific serovar is formally demonstrated.

## Concluding remarks

Serotyping has been the reference method for *Leptospira* typing for over 50 years. However, most serotyping methods are complex and now obsolete. They also have a number of drawbacks, including high costs and the need for highly trained staff. In addition, the current serotyping methods may not distinguish strains with different properties within a single serovar and closely related serovars; this may be of importance in epidemiological studies, surveillance, diagnostics, vaccine development and host–pathogen interactions. There is, therefore, an urgent need for alternative methods, including molecular typing methods that accurately reflect the diversity of *Leptospira* isolates.

The main advantages of DNA-based approaches are their accessibility, low cost, robustness and the availability of publicly accessible databases that allow comparison of data from laboratories worldwide. Previous phylogenetic analysis revealed that most serogroups had a polyphyletic distribution and did not descend from a single ancestor [18]. However, we and others found that the *rfb* cluster polymorphism was strongly correlated with serovar [12,35,40]. Recently developed methods such as targeted DNA capture and AmpSeq [76,78] offer promising tools for genotyping directly from biological and environmental samples, bypassing the need for culture isolation. Using this locus for molecular typing is a promising alternative that will be critical to detecting outbreaks early and identifying their sources.

## Supplementary material

10.1099/jmm.0.002059 Uncited Table S1.

## Abbreviations

CAAT: cross-agglutinin absorption test
CGs: clonal groups
LPS: lipopolysaccharides
MAT: microscopic agglutination testing
MLST: multilocus sequence typing
WGS: whole-genome sequencing
