# Supplementary material for: Shaping the future of Leptospira serotyping
Source: J Med Microbiol. 2025 Sep 12;74(9):002059. doi: 10.1099/jmm.0.002059 (PMC12451757; doi:10.1099/jmm.0.002059)
Supplement: Uncited Table S1. [file jmm-74-02059-s001.pdf]

Table S1: List of leptospiral antigen used for MAT at the french National Reference Center for Leptospirosis

Species	Serogroup	Serovar	Strain
L. interrogans	Australis	Australis	Ballico
L. interrogans	Autumnalis	Autumnalis	Akiyami A
L. interrogans	Bataviae	Bataviae	Van Tienen
L. interrogans	Canicola	Canicola	Hond Utrecht IV
L. borgpetersenii	Ballum	Castellonis	Castellon 3
L. kirschneri	Cynopteri	Cynopteri	3522 C
L. kirschneri	Grippotyphosa	Grippotyphosa	Moskva V
L. interrogans	Sejroe	Hardjobovis	Sponselee
L. interrogans	Hebdomadis	Hebdomadis	Hebdomadis
L. interrogans	Icterohaemorrhagiae	Copenhageni	Wijnberg
L. noguchii	Panama	Panama	CZ 214 K
L. biflexa	Semaranga	Patoc	Patoc 1
L. interrogans	Pomona	Pomona	Pomona
L. interrogans	Pyrogenes	Pyrogenes	Salinem
L. borgpetersenii	Sejroë	Sejroë	M 84
L. borgpetersenii	Tarassovi	Tarassovi	Mitis Johnson
L. interrogans	Icterohaemorrhagiae	Icterohaemorrhagiae	Verdun
L. weilii	Celledoni	ND	2011/01963
L. interrogans	Djasiman	Djasiman	Djasiman
L. borgpetersenii	Mini	ND	2008/01925
L. weilii	Sarmin	Sarmin	Sarmin
L. santarosai	Shermani	Shermani	1342 K
L. borgpetersenii	Javanica	Javanica	Poi
L. noguchii	Louisiana	Louisiana	LUC1945

ND: Non determined
